# Dynamics of Mental Disorders After Combat Brain Injuries in The Acute and Remote Periods

**DOI:** 10.1192/j.eurpsy.2026.11332

**Published:** 2026-07-31

**Authors:** V. Kuzminov, I. Linskiy, V. Zadorozhny, R. Lakinskiy, M. Denysenko, T. Tkachenko, O. Minko

**Affiliations:** 1 Department of emergency psychiatry and narcology; 2 Department of emergency psychiatry and narcology, SI “Institute of Neurology, Psychiatry and Narcology named after P.V. Voloshin of the NAMS of Ukraine”, Kharkiv, Ukraine

## Abstract

**Introduction:**

An accurate assessment of the mental state of the patient in the conditions of hostilities and in the remote period is a prerequisite for the timely provision of medical and rehabilitation assistance.

**Objectives:**

The purpose of the study. Develop an algorithm for assessing the condition of a patient with traumatic brain injury and the course of mental disorders at different stages of a traumatic brain injury, taking into account the dynamic changes in the patient’s condition. Materials. Patients with mental disorders who suffered a closed craniocerebral injury in the conditions of hostilities.

**Methods:**

Clinical-psychopathological, anamnestic, study of medical documentation.

**Results:**

The possibility of changes from non-psychotic variants of impaired consciousness to an acute psychotic state was established immediately after a craniocerebral injury. Most patients reported a period of imaginary wellbeing or psychomotor excitement in the conditions of hostilities after a craniocerebral injury. On the one hand, this made it possible to survive in the conditions of a combat encounter, on the other hand, it delayed the provision of qualified medical assistance. Factors studied in the development of psychotic forms of impaired consciousness in the acute period of craniocerebral injury. The general patterns characteristic of all types of traumatic brain damage are presented as follows. The acute period - the suddenness of the lesion, which causes a maximum of pathological changes immediately after the craniocerebral injury; the regression of the further development of painful phenomena (from severe to milder) in the acute and intermediate periods; the formation of new symptoms of the disease in connection with the growth of the scar (in case of contusion) or involving new foci in the process; persistence of psychopathological disorders in the remote period of craniocerebral injury. Thus, the main difference between the intermediate and remote period is the possibility of reducing the manifestations of brain damage and preventing the development of some cointermediate and distant period. They turned out to be the presence of a mental illness before the injury, addiction to psychoactive substances, acute stress, other accompanying somato-neurological diseases. In the long-term period of craniocerebral injury, depending on the severity, persistent various organic mental disorders were observed. Deterioration of the mental state (primarily cognitive abilities) in the remote period was associated with repeated craniocerebral injury, addiction to psychoactive substances, other toxic factors, accumulated diseases during life.

**Conclusions:**

Mental disorders in craniocerebral injuries are dynamic, which must be considered during the initial assessment of the patient’s condition and during further planning of treatment and rehabilitation.

**Disclosure of Interest:**

None Declared

